# Supplementary material for: Persistent symptoms after COVID-19 are not associated with differential SARS-CoV-2 antibody or T cell immunity
Source: Nat Commun. 2023 Aug 23;14:5139. doi: 10.1038/s41467-023-40460-1 (PMC10447583; doi:10.1038/s41467-023-40460-1)
Supplement: Supplementary file 1 — Supplementary Information [file 41467_2023_40460_MOESM1_ESM.pdf]

Supplementary Information

Persistent symptoms after COVID-19 are not associated with differential SARS-CoV-2 antibody or T cell immunity

Daniel M. Altmann[1*], Catherine J Reynolds[2], George Joy[3,4], Ashley D. Otter[5], Joseph M. Gibbons[6], Corinna Pade[6], Leo Swadling[7], Mala K. Maini[7], Tim Brooks[5], Amanda Semper[5], Áine McKnight[6], Mahdad Noursadeghi[7], Charlotte Manisty[3,4], Thomas A Treibel[3,4], James C Moon[3,4], COVIDsortium investigators[$], Rosemary J Boyton[2,8*]

[1]Department of Immunology and Inflammation, Imperial College London, London, UK
[2]Department of Infectious Disease, Imperial College London, London, UK
[3]St Bartholomew's Hospital, Barts Health NHS Trust, London, UK
[4]Institute of Cardiovascular Science, University College London, UK
[5]UK Health Security Agency, Porton Down, UK
[6]Blizard Institute, Barts and the London School of Medicine and Dentistry, Queen Mary University of London, London, UK
[7]Division of Infection and Immunity, University College London, London, UK
[8]Lung Division, Royal Brompton and Harefield Hospitals, Guy's and St Thomas' NHS Foundation Trust, London, UK

*Corresponding authors
Prof. Rosemary Boyton, PhD, FRCP, FHEA
Prof. Daniel Altmann, PhD
Faculty of Medicine, Hammersmith Hospital Campus
Imperial College London, Du Cane Road, London W12 0NN, UK.
Email: r.boyton@imperial.ac.uk; d.altmann@imperial.ac.uk

Supplementary Figure 1. Longitudinal S1 RBD antibody titers (black circle) and T cell responses against spike megapool (purple circle). Longitudinal data is shown from the date of SARS-CoV2 PCR positive confirmed infection and over the following 15 week follow up for individual HCW. Source data are provided as a Source Data file. (**A**) HCW that had Recovered 12 months after infection (closed circle) and (**B**) HCW reporting Persistent symptoms at 12 months (open circle). Ab, antibody; HCW, health care worker; PBMC, peripheral blood mononuclear cells; RBD, receptor binding domain; SFC, spot forming cells; U, units.

A

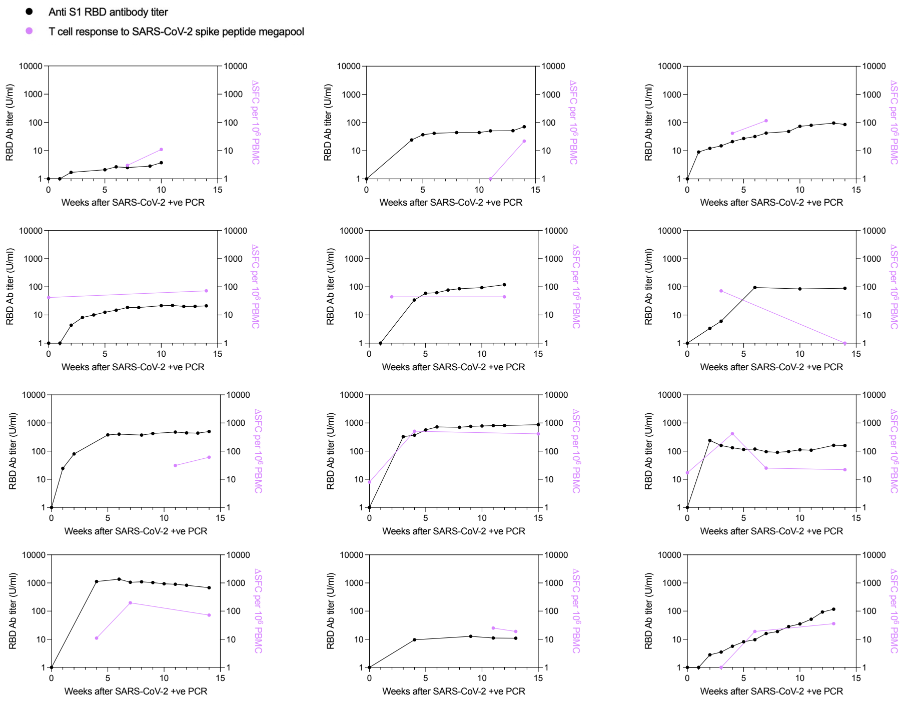

B

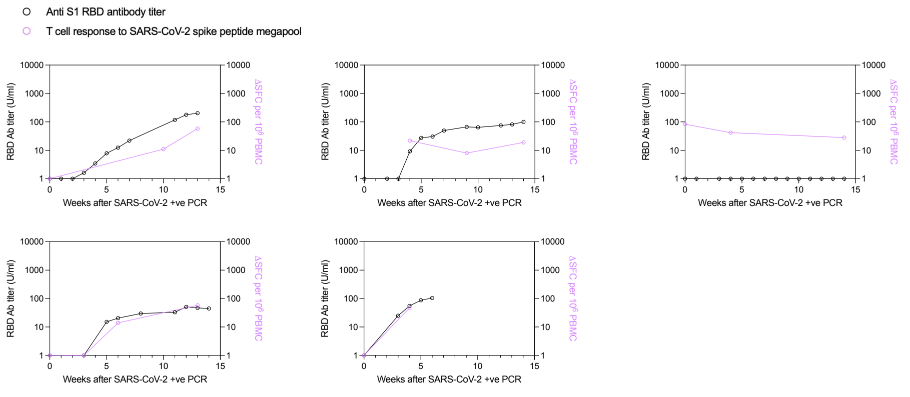

Supplementary Figure 2. Longitudinal (**A**) S1 RBD and (**B**) nucleocapsid antibody titers measured in the whole HCW cohort (n=86, 33% male: Recovered, closed circle, n = 61, 34% male; Persistent symptoms, open circle, n = 25, 28% male) are annotated to indicate HCW (Recovered, orange closed circle, n = 15, 57% male; Persistent symptoms, orange open circle, n = 7, 57% male) that were included in the neutralizing antibody and T cell assays at 12-month follow-up. Source data are provided as a Source Data file. Two-tailed Mann-Whitney U tests (Graphpad Prism version 8.0) were used to test for significant differences between recovered HCW and those reporting persistent symptoms. Error bars shown are geometric mean ± 1 geometric SD. Ab, antibody; COI, cut-off index; HCW, health care worker; N, nucleocapsid; RBD, receptor binding domain; SD, standard deviation; U, units.

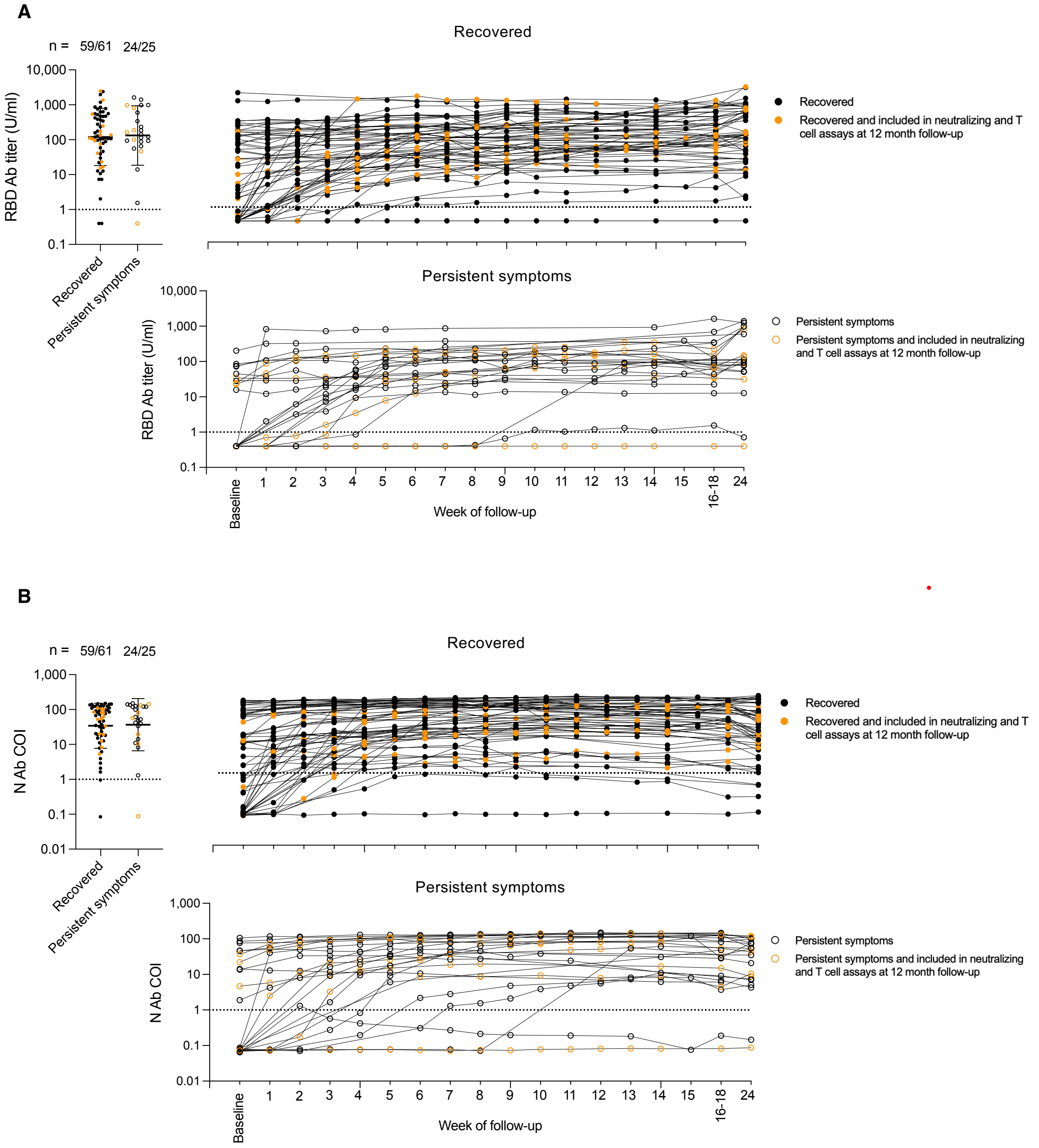

Supplementary Figure 3. CONSORT diagram for Persistent symptom and Recovered sub-study recruitment including assays performed at each timepoint

COVIDsortium cohort recruited HCW from St Bartholomew's, Nightingale and Royal Free Hospital NHS Hospitals, London between 23rd March and 7th May 2020

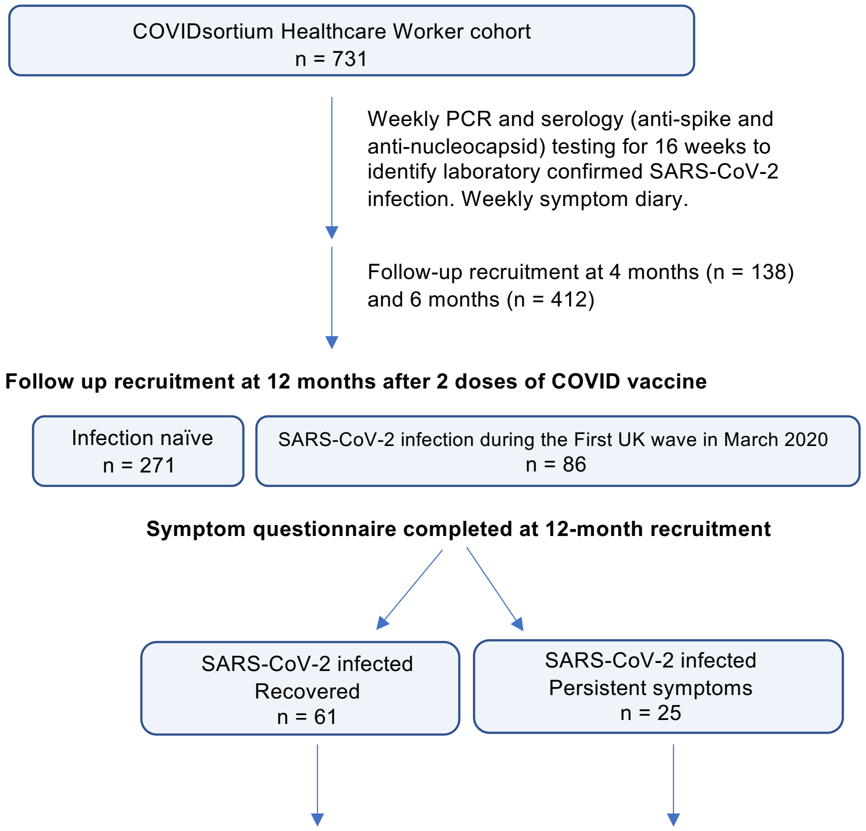

Baseline to 4 months

Data analysis	*n =*	*n =*
S1 RBD serology	61	25
Nucleocapsid serology	61	25
Pseudovirus neutralization	37	21
Protein and MEP pool T cell ELISpot	37	21
FEC peptide ELISpot	34	19
Peptide megapool T cell ELISpot	14	8

12 months

Data analysis	*n =*	*n =*
S1 RBD serology	53	25
Nucleocapsid serology	61	25
Live virus neutralization	14	7
MEP pool T cell ELISpot	14	7

COVIDsortium Investigators:

Hakam Abbass[3], Aderonke Abiodun[3,4], Mashael Alfarih[3], Zoe Alldis[3], Daniel M Altmann[1], Oliver E Amin[7], Mervyn Andiapen[3], Jessica Artico[3], João B Augusto[3], Georgina L Baca[3], Sasha N L Bailey[2], Anish N Bhuva[3], Alex Boulter[3], Ruth Bowles[3], Rosemary J Boyton[2,8], Olivia V Bracken[9], Ben O'Brien[3,10], Tim Brooks[5], Natalie Bullock[6], David K Butler[2], Gabriella Captur[4,11], Olivia Carr[5], Benjamin M Chain[7], Nicola Champion[3], Carmen Chan[3], Aneesh Chandran[7], David Collier[20], Tom Coleman[5], Jorge Couto de Sousa[3], Xose Couto-Parada[3], Eleanor Cross[5], Teresa Cutino-Moguel[3], Silvia D'Arcangelo[5], Rhodri H Davies[3], Brooke Douglas[11], Cecilia Di Genova[14], Keenan Dieobi-Anene[3], Mariana O Diniz[7], Anaya Ellis[5], Karen Feehan[9], Malcolm Finlay[3], Marianna Fontana[11], Nasim Forooghi[3], Sasha Francis[5], Celia Gaier[9], Joseph M Gibbons[6], David Gillespie[3], Derek Gilroy[15], Matt Hamblin[3], Gabrielle Harker[5], Georgia Hemingway[5], Jacqueline Hewson[5], Wendy Heywood[16,17], Lauren M Hickling[18], Bethany Hicks[5], Aroon D Hingorani[7], Lee Howes[4], Alun Hughes[4], Gemma Hughes[3], Rebecca Hughes[3,4], Ivie Itua[3], Victor Jardim[3], Wing-Yiu Jason Lee[6], Melanie Petra Jensen[3], Jessica Jones[5], Meleri Jones[6], George Joy[3,4], Vikas Kapil[3,20], Caoimhe Kelly[5], Hibba Kurdi[3,4], Jonathan Lambourne[3], Kai-Min Lin[2], Siyi Liu[2], Aaron Lloyd[5], Sarah Louth[11], Mala K Maini[7], Vineela Mandadapu[3], Charlotte Manisty[3,4], Áine McKnight[6], Katia Menacho[3], Celina Mfuko[3], Kevin Mills[16,17], Sebastian Millward[5], Oliver Mitchelmore[3], Christopher Moon[5], James C Moon[3,4], Diana Muñoz Sandova[2], Sam M Murray[2], Mahdad Noursadeghi[7], Ashley Otter[5], Corinna Pade[6], Susana Palma[3], Ruth Parker[19], Kush Patel[3], Mihaela Pawarova[11], Steffen E Petersen[3], Brian Piniera[3], Franziska P Pieper[2], Daniel Pope[3,20], Mary Prossora[3], Lisa Rannigan[11], Alicja Rapala[4], Catherine J Reynolds[2], Amy Richards[3], Matthew Robathan[20], Joshua Rosenheim[7], Cathy Rowe[5], Matthew Royds[5], Jane Sackville West[21], Genine Sambile[3], Nathalie M. Schmidt[7], Hannah Selman[5], Amanda Semper[5], Andreas Seraphim[3], Mihaela Simion[11], Angelique Smit[11], Michelle Sugimoto[9], Leo Swadling[7], Stephen Taylor[5], Nigel Temperton[14], Stephen Thomas[5], George D Thornton[3,4], Thomas A Treibel[3,4], Art Tucker[3], Ana M Valdes[22,23], Ann Varghese[5], Jessry Veerapen[3], Mohit Vijayakumar[3], Sophie Welch[3], Hannah White[5], Theresa Wodehouse[3], Lucinda Wynne[3], and Dan Zahedi[19]

[1]Department of Immunology and Inflammation, Imperial College London, London, UK
[2]Department of Infectious Disease, Imperial College London, London, UK
[3]St Bartholomew's Hospital, Barts Health NHS Trust, London, UK
[4]Institute of Cardiovascular Science, University College London, UK
[5]UK Health Security Agency, Porton Down, UK
[6]Blizard Institute, Barts and the London School of Medicine and Dentistry, Queen Mary University of London, London, UK
[7]Division of Infection and Immunity, University College London, London, UK
[8]Lung Division, Royal Brompton and Harefield Hospitals, Guy's and St Thomas' NHS Foundation Trust, London, UK
[9]Division of Medicine, University College London, London, UK
[10]German Heart Centre and Charité University, Berlin, Germany
[11]Royal Free London NHS Foundation Trust, London, UK
[14]Viral Pseudotype Unit, Medway School of Pharmacy, Chatham Maritime, Kent, UK
[15]Centre for Clinical Pharmacology, University College London, London, UK
[16]Translational Mass Spectrometry Research Group, Genetics & Genomic Medicine, UCL Institute of Child Health, London, UK
[17]Great Ormond Street Biomedical Research Centre, UCL Institute of Child Health, London, UK
[18]East London NHS Foundation Trust Unit for Social and Community Psychiatry, Newham Centre for Mental Health, London, UK
[19]School of Clinical Medicine, University of Cambridge, Cambridge, UK
[20]William Harvey Research Institute, Queen Mary University of London, London, UK
[21]James Wigg Practice, Kentish Town, London, UK
[22]Academic Rheumatology, Clinical Sciences, Nottingham City Hospital, Nottingham, UK
[23]NIHR Nottingham Biomedical Research Centre, Nottingham University Hospitals NHS Trust and University of Nottingham, Nottingham, UK

[Professor James C Moon is the UK COVIDsortium Investigators representative: email: james.moon1@nhs.net]
