# Supplementary material for: Persistent symptoms after COVID-19 are not associated with differential SARS-CoV-2 antibody or T cell immunity
Source: Nat Commun. 2023 Aug 23;14:5139. doi: 10.1038/s41467-023-40460-1 (PMC10447583; doi:10.1038/s41467-023-40460-1)
Supplement: Supplementary file 3 — Reporting Summary [file 41467_2023_40460_MOESM3_ESM.pdf]

nature portfolio

Corresponding author(s): Altmann DM & Boyton RJ

Last updated by author(s): Jul 11, 2023

Reporting Summary

Nature Portfolio wishes to improve the reproducibility of the work that we publish. This form provides structure for consistency and transparency in reporting. For further information on Nature Portfolio policies, see our Editorial Policies and the Editorial Policy Checklist.

Statistics

For all statistical analyses, confirm that the following items are present in the figure legend, table legend, main text, or Methods section.

n/a	Confirmed	
☐	☒	The exact sample size (*n*) for each experimental group/condition, given as a discrete number and unit of measurement
☐	☒	A statement on whether measurements were taken from distinct samples or whether the same sample was measured repeatedly
☐	☒	The statistical test(s) used AND whether they are one- or two-sided<br>*Only common tests should be described solely by name; describe more complex techniques in the Methods section.*
☒	☐	A description of all covariates tested
☐	☒	A description of any assumptions or corrections, such as tests of normality and adjustment for multiple comparisons
☐	☒	A full description of the statistical parameters including central tendency (e.g. means) or other basic estimates (e.g. regression coefficient) AND variation (e.g. standard deviation) or associated estimates of uncertainty (e.g. confidence intervals)
☒	☐	For null hypothesis testing, the test statistic (e.g. *F*, *t*, *r*) with confidence intervals, effect sizes, degrees of freedom and *P* value noted<br>*Give P values as exact values whenever suitable.*
☒	☐	For Bayesian analysis, information on the choice of priors and Markov chain Monte Carlo settings
☒	☐	For hierarchical and complex designs, identification of the appropriate level for tests and full reporting of outcomes
☒	☐	Estimates of effect sizes (e.g. Cohen's *d*, Pearson's *r*), indicating how they were calculated

Our web collection on statistics for biologists contains articles on many of the points above.

Software and code

Policy information about availability of computer code

Data collection	No software was used in data collection.
Data analysis	Prism V 8.0 for Mac software was used for data analysis

For manuscripts utilizing custom algorithms or software that are central to the research but not yet described in published literature, software must be made available to editors and reviewers. We strongly encourage code deposition in a community repository (e.g. GitHub). See the Nature Portfolio guidelines for submitting code & software for further information.

Data

Policy information about availability of data

All manuscripts must include a data availability statement. This statement should provide the following information, where applicable:

- Accession codes, unique identifiers, or web links for publicly available datasets
- A description of any restrictions on data availability
- For clinical datasets or third party data, please ensure that the statement adheres to our policy

All data needed to evaluate the conclusions in the paper are presented in the paper or the Supplementary Information. Source data are provided with this paper.

Research involving human participants, their data, or biological material

Policy information about studies with human participants or human data. See also policy information about sex, gender (identity/presentation), and sexual orientation and race, ethnicity and racism.

Reporting on sex and gender	Adult Healthcare workers HCW (>18 years old) self-declared as fit to attend work. Mean age of the COVIDsortium cohort (n=731) was 38±11 years; 33% male were recruited at the start of the study in March 2020.<br>At 4 months 58 HCW (mean age 42y, 28% male) were recruited with laboratory confirmed SARS-CoV-2 infection either by SARS-CoV-2 positive PCR and/or positive for spike IgG (Euroimmun ELISA) / N IgG/IgM antibody (Roche Elecsys).<br>At 6 months 399 HCW were recruited. 308 were infection naive (mean age 39y, 31% male). 91 had laboratory confirmed SARS-CoV-2 infection during the first wave. Of these, 75 had fully recovered (mean age 4ly, 37% male) and 16 had persistent symptoms (mean age 38y, 31% male) at 6 months f/u. [Table 1]<br>A total of n=357 HCW were recrutied at 55-57-week follow-up (mean age 39y, 32% male). 271 HCW were infection naive (mean age 40y, 34% male). Eighty-six had laboratory confirmed SARS-CoV-2 infection (Wuhan Hu-1) during the first wave. Of these, 61 had fully recovered (mean age 42y, 34% male) and 25 had persistent symptoms (mean age 39y, 28% male) at 12 month f/u. [Table 2]
Reporting on race, ethnicity, or other socially relevant groupings	All Adult Healthcare workers HCW (>18 years old). At baseline, 37% of participants were Black, Asian or minority ethnicities (BAME)
Population characteristics	Adult UK NHS Healthcare workers HCW (>18 years old) working in London and self-declared as fit to attend work were enrolled into the study in March 2020 and invited for longitudinal follow-up. Mean age of the COVIDsortium cohort (n=731) was 38±11 years; 33% male were recruited at the start of the study in March 2020.
Recruitment	Adult Healthcare workers HCW (>18 years old) self-declared as fit to attend work were invited to be enrolled in this observational, longitudinal follow-up HCW study. There was no obvious identifiable self-selection bias. All HCW recruited with laboratory confirmed SARS-CoV-2 infection (by positive PCR and/or Sl-RBD / N serology) during the first wave that presented for f/u, had blood drawn, completed symptom diaries / questionnaires and had received two Pfizer COVID vaccines before the 12 month recruitment were included in this study analysis.<br>HCW completed questionnaires exploring demographic, clinical and exposure risks, and samples were collected at baseline and weekly follow-up for 16-18w from the start. Participants were asked to provide details and timing of symptoms in the 3 months prior to baseline, and for those unable to attend follow-up visits (due to shift rostering, annual leave or self-isolation), the reason for non-attendance was collected, to ensure capture of information regarding self-isolation due to participant symptoms or household contacts. On return from self-isolation with symptoms, convalescent samples were collected. HCW were invited for further follow-up at 6, 12 months.
Ethics oversight	The COVIDsortium Healthcare Workers bioresource was approved by the ethical committee of UK National Research Ethics Service (20/SC/0149) and registered on ClinicalTrials.gov (NCT04318314). The study conformed to the principles of the Helsinki Declaration, and all subjects gave written informed consent.

Note that full information on the approval of the study protocol must also be provided in the manuscript.

Field-specific reporting

Please select the one below that is the best fit for your research. If you are not sure, read the appropriate sections before making your selection.

☒ Life sciences ☐ Behavioural & social sciences ☐ Ecological, evolutionary & environmental sciences

For a reference copy of the document with all sections, see nature.com/documents/nr-reporting-summary-flat.pdf

Life sciences study design

All studies must disclose on these points even when the disclosure is negative.

Sample size	All HCW recruited at each time point with laboratory confirmed SARS-CoV-2 infection during the first wave that presented for f/u and completed symptom diaries / questionnaires and had received two Pfizer COVID vaccines before the 12 month recruitment were included.
Data exclusions	Adult Healthcare workers HCW (>18 years old) who self-declared as fit to attend work were invited to participate via local advertisement of the project (see https://covid-consortium.com).
Replication	The number of times an experiment was replicated in the laboratory is recorded in the Methods that describes each in vitro experiment. n numbers are stated on Figures and in figure legends for Figure 1 & Figure 2 and Supplementary Figure 1 & 2. Samples analysed in the study were taken from all participants recruited at a particular time point and samples were analysed from individual HCW participants. Experiments did not include replicates as all HCW participants and data points are unique. All the experiments shown in Figure 1 & Figure 2 and Supplementary Figure 1 & Supplementary Figure 2 were all preformed using technical duplicates.

Randomization: Randomization was not appropriate for this study as there was no therapeutic intervention.

Blinding: Blinding was not appropriate for this study as there was no therapeutic intervention. The laboratory staff were blinded to HCW ID when doing experimental work.

Reporting for specific materials, systems and methods

We require information from authors about some types of materials, experimental systems and methods used in many studies. Here, indicate whether each material, system or method listed is relevant to your study. If you are not sure if a list item applies to your research, read the appropriate section before selecting a response.

Materials & experimental systems

n/a	Involved in the study	
[ ]	[x]	Antibodies
[ ]	[x]	Eukaryotic cell lines
[x]	[ ]	Palaeontology and archaeology
[x]	[ ]	Animals and other organisms
[ ]	[x]	Clinical data
[x]	[ ]	Dual use research of concern
[x]	[ ]	Plants

Methods

n/a	Involved in the study	
[x]	[ ]	ChIP-seq
[x]	[ ]	Flow cytometry
[x]	[ ]	MRI-based neuroimaging

Antibodies

Antibodies used

T cell ELISpot antibodies:
- Anti-human IFNg mAb, clone 1-D1K, Mabtech, #3420-3, pre-coated on plates supplied by Mabtech.
- Alkaline phosphatase conjugated anti-human IFNg mAb, clone 7-B6-1ALP, Mabtech, #3420-9A, used at 1:200
- Anti-CD3 mAb, clone CD3-2, Mabtech, #3605-1-50, used at 1:1000

Viral titer antibody:
- Goat anti-human IgG -galactosidase-conjugated, Polyclonal, Southern Biotech, #2040-06, used at 1:400

Validation

Anti-human IFNg mAb, clone 1-D1K
https://stella.mabtech.com/sites/default/files/product_datasheets/3420-3-250.pdf
Validated for use in ELISpot assays

Alkaline phosphatase conjugated anti-human IFNg, clone 7-B6-1ALP
https://stella.mabtech.com/sites/default/files/product_datasheets/3420-9A-1000.pdf
Validated for use in ELISpot assays

Anti-CD3, clone CD3-2
https://stella.mabtech.com/sites/default/files/product_datasheets/3605-1-50.pdf
Validated for use in ELISpot assays

Goat anti-human IgG -galactosidase-conjugated: Polyclonal
https://resources.southernbiotech.com/techbul/2040.pdf
Quality tested applications include ELISA, FLISA and Flow cytometry.

Eukaryotic cell lines

Policy information about cell lines and Sex and Gender in Research

Cell line source(s)

VeroE6 (ATCC, The Global Bioresource Centre, #VERO C1008)

HEK293T (ATCC, The Global Bioresource Centre, #CRL-3216)

HuH7 (ECACC, European Collection of Authenticated Cell Cultures, #01042712)

Authentication: Cell lines were authenticated by ATCC and ECACC

Mycoplasma contamination: All cell lines were regularly tested for mycoplasma and tested negative throughout.

Commonly misidentified lines (See ICLAC register): No commonly misidentified lines were used in this study.

Clinical data

Policy information about clinical studies

All manuscripts should comply with the ICMJE guidelines for publication of clinical research and a completed CONSORT checklist must be included with all submissions.

Clinical trial registration	The COVIDsortium Healthcare Workers bioresource was approved by the ethical committee of UK National Research Ethics Service (20/SC/0149) and registered on ClinicalTrials.gov (NCT04318314). The study conformed to the principles of the Helsinki Declaration, and all subjects gave written informed consent.
Study protocol	J. B. Augusto, K. Menacho, M. Andiapen, R. Bowles, M. Burton, S. Welch, A. Bhuva, A. Seraphim, C. Pade, G. Joy, M. Jensen, R. H. Davies, G. Captur, M. Fontana, H. Montgomery, B. O'Brien, A. D. Hingorani, T. Cutino-Moguel, A. McKnight, H. Abbass, M. Alfarih, Z. Alldis, G. Baca, A. Boulter, 0. Bracken, N. Bullock, N. Champion, C. Chan, X. Couto-Parada, K. Dieobi-Anene, K. Feehan, G. Figtree, M. C. Figtree, M. Finlay, N. Forooghi, J.M. Gibbons, P. Griffiths, M. Hamblin, L. Howes, I. ltua, M. Jones, V. Jardim, V. Kapil, W.-Y. Jason Lee, V. Mandadapu, C. Mfuko, 0. Mitchelmore, S. Palma, K. Patel, S. E. Petersen, B. Piniera, R. Raine, A. Rapala, A. Richards, G. Sambile, J. Couto de Sousa, M. Sugimoto, G. D. Thornton, J. Artico, D. Zahedi, R. Parker, M. Robathan, L. M. Hickling, N. Ntusi, A. Semper, T. Brooks, J. Jones, A. Tucker, J. Veerapen, M. Vijayakumar, T. Wodehouse, L. Wynne, T. A. Treibel, M. Noursadeghi, C. Manisty, J. C. Moon, Healthcare Workers Bioresource: Study outline and baseline characteristics of a prospective healthcare worker cohort to study immune protection and pathogenesis in COVID-19. Wellcome Open Res. 5, 179 (2020).
Data collection	A prospective, observational, longitudinal cohort design was used. A cohort of 400 HCW was initially recruited from St Bartholomew's Hospital, London, in the week of UK lockdown (23rd-31st March 2020). All participants were asymptomatic and self-declared fit to attend work in hospital. Recruitment was extended (27th April-7th May 2020) to include 331 additional participants from multiple sites: St Bartholomew's Hospital (n=l0l additional), NHS Nightingale Hospital (n=l0), and Royal Free NHS Hospital Trust (n=220). The study protocol consisted of asking HCW to complete questionnaires exploring demographic, clinical and exposure risks, and sample collection at baseline and weekly follow-up for 16-18w from the start of each cohort. Participants were asked to provide details and timing of symptoms in the 3 months prior to baseline, and for those who were unable to attend follow-up visits (due to shift rostering, annual leave or self-isolation), the reason for non-attendance was collected, to ensure capture of information regarding isolation due to participant symptoms or household contacts. On return from self-isolation with symptoms, convalescent samples were collected. HCW participants were invited to attend further longitudinal follow-ups visits at 6, and 12 months. In London, the case-doubling time in March, 2020 was approximately 3-4 days. The number of nasal swabs testing positive for SARS-CoV-2 in our study peaked at March 23rd to 31st, 2020 suggesting that infections peaked on or around March 23rd, 2020, the day of UK lockdown. We thus observed approximately synchronous infections coincident with the peak epidemic transmission in London at the start of the study, UK lockdown on March 23rd and therefore used this as the benchmark starting point for our analysis of T cell and nAb responses in the first wave.
Outcomes	A prospective, observational, longitudinal cohort design was used. There were no pre-defined primary or secondary outcomes.
